# Cervical cancer screening: Impact of collection technique on human papillomavirus detection and genotyping

**DOI:** 10.1016/j.pmedr.2025.102971

**Published:** 2025-01-17

**Authors:** Alisa P. Young, Mutiya Olorunfemi, Leigh Morrison, Scott A. Kelley, Anna Laurie, Anna McEvoy, Jill Schneiderhan, Julie Prussack, Marie Claire O'Dwyer, Pamela Rockwell, Philip Zazove, Jonathan Gabison, Jane Chargot, Kristina Gallagher, Ananda Sen, Dongru Chen, Elizabeth A. Haro, Emma A. Butcher, Martha L. Alves, Christelle El Khoury, Melinda L. Dendrinos, Nicole Brashear, Roger Smith, Richard W. Lieberman, Natalie Saunders, Elizabeth Campbell, Heather M. Walline, Diane M. Harper

**Affiliations:** a Department of Family Medicine, University of Michigan Medical School, 300 North Ingalls Street, NI4C06, Ann Arbor, MI 48109, USA; b Department of Obstetrics and Gynecology, 1500 E Medical Center Dr # 9, University of Michigan Medical School, Ann Arbor, MI 48109, USA; c Department of Otolaryngology, 1500 E Medical Center Dr # 1 University of Michigan Medical School, Ann Arbor, MI 48109, USA; d Department of Biostatistics, School of Public Health, 1415 Washington Heights, University of Michigan, Ann Arbor, MI 48109, USA

**Keywords:** Cervical cancer screening, Self-sampling., Primary HPV screening., Agreement., Kappa., Sensitivity., Specificity.

## Abstract

**Background:**

The Food and Drug Administration (FDA) in the US approved primary human papillomavirus (HPV) testing for speculum-based cervical cancer screening ten years ago and, in May 2024, approved the self-collection technique. Our study defines the kappa agreement between self- and speculum-based collection techniques for 15 types of high-risk HPV. Additionally, we describe the sensitivity and specificity ratios for HPV testing using both collection methods.

**Methods:**

Participants recruited in 2020–2022 included 97 colposcopy attendees and 96 routine primary care screening attendees aged 30–65, who agreed to self-sample before their clinically scheduled speculum-based exam. Prevalence-based kappa calculated agreement, sensitivity and specificity ratios calculated accuracy using the cervical intraepithelial neoplasia grade 2 or worse (CIN 2+) threshold.

**Results:**

The average ages were 45.9 (SD 10.5) and 46.2 (SD 11.0) years for the colposcopy and primary care attendees, respectively. HPV 16, 68, and 39 were the most common types detected. The lower bound of the 95 % Cl for kappa calculations was above 0.81, indicating almost perfect agreement across all HPV genotypes. The sensitivity and specificity ratios were consistent at 1.0 across both collection methods. The HPV positivity rate was significantly higher among colposcopy attendees at 66 % (64/97), compared to 14 % (13/96) among routine primary care screeners. The study identified 17 women with CIN2 + .

**Conclusions:**

Primary HPV screening with self-collection is equivalent to speculum-based collection among people aged 30–65. The findings emphasize the utility of self-collection in identifying high-grade lesions and the consistency of HPV detection across different collection methods.

## Highlights

• Human papillomavirus prevalence is 66 % among colposcopy attendees.
• Human papillomavirus prevalence is 14 % among routine primary care screeners.
• Self-sampling is equivalent to speculum-based human papillomavirus detection.
• Self is equal to speculum for Cervical Intraepithelial Neoplasia grade 2 or worse.

## 1 Introduction

Meta-analyses on primary human papillomavirus (HPV)-based cervical cancer screening assessed HPV collection techniques evaluated using concordance data from 59 diagnostic test accuracy studies and 26 randomized controlled trials (RCTs), none of which occurred on US-based screening-eligible populations(Arbyn et al., 2022; Arbyn et al., 2018b). The United States falls behind other countries in evidence-based data to show that speculum-based and self-collection techniques do not differ in HPV detection. Likewise, no US-based large data evaluates primary HPV detection through a self or speculum-based collection technique as a predictor for cervical intraepithelial neoplasia grade2 or worse (CIN 2+) disease.

Moreover, the US clinician community has not embraced updated strategies to decrease the incidence of cervical cancer (Dorismond et al., 2024). At the same time, over the past 20 years, the incidence of cervical cancer in the US has not varied in any meaningful way from 8/100,000 screening-eligible persons (Centers for Disease Control, 2024), twice the rate of the WHO Cervical Cancer Elimination goal (WHO, 2020). Countries that have adopted primary HPV testing and included self-screening have reduced their cervical cancer incidence (Hall et al., 2019; Hellsten et al., 2024; Horn et al., 2019; Wilailak et al., 2021) and have reached those who have not otherwise participated in screening (Polman et al., 2019; Verdoodt et al., 2015).

Primary HPV screening for cervical cancer has been approved by the Food and Drug Administration (FDA) for ten years in the US (Roche, 2014), with two more platforms approved for speculum-based testing in 2018 and 2023 (FDA, 2019, PR Newswire, 2023). Two FDA-approved HPV testing platforms for in-office self-sampling (Roche, 2024; BD, 2024) were approved this year 2024.

We aim to define the accuracy/agreement for HPV detection between self and speculum-based collection techniques for 15 types of high-risk HPV in a well-screened US-based population of two cohorts: those whose risk for CIN 3+ is greater than 4 % and those who present for routine screening. Secondarily, we aim to define the sensitivity and specificity and their ratios against a clinical CIN 2+ threshold for HPV testing by self and speculum-based techniques.

## 2 Methods

### 2.1 Population recruitment

We recruited people whose risk of CIN 3+ was greater than 4 % and were referred to a colposcopy clinic as our high-prevalence HPV population. In addition, we recruited people from primary care screening to identify a population with the general prevalence of HPV infection. Other inclusion criteria were age between 30 and 65 years, ability to use a vaginal sampling device, not pregnant, not menstruating at the time of collection, not immunocompromised, having no current cancer or self-reported poor health.

**Fig. 1:**
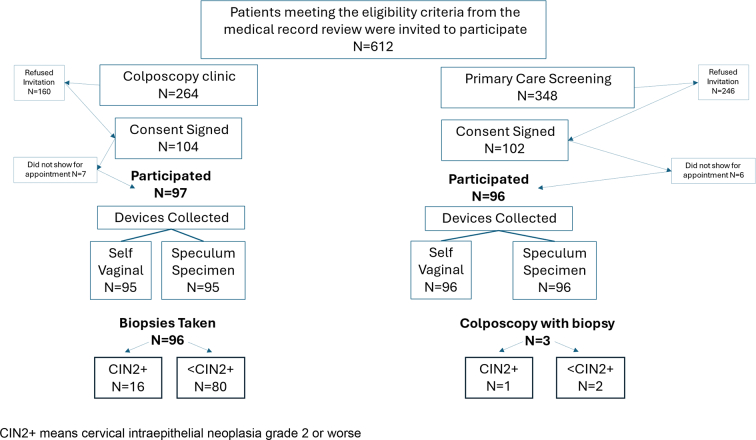
US based study of high and average risk asymptomatic women enrolled in device collection trial to detect cervical intraepithelial neoplasia grade 2 or worse (CIN2+), 2020–2022.

We reviewed all appointments for patients enrolled at the University of Michigan Health System weekly between October 2020 and February 2022 to ascertain eligible candidates in both cohorts. The study team contacted each patient before their appointments to invite study participation and consent. We consented consecutive participants until we reached 100 persons in each cohort. Not all participants came for their appointments. [Fig. 1]. A total of 193 people participated.

### 2.2 Collection techniques and devices

We block-randomized all enrolled persons to receive one of two possible vaginal sampling devices: a dacron swab with pre-scoring on the shaft and the HerSwab device®. We chose the first device for its simplicity and the second because it provided field-tested evidence for maximum comfort in the collection. We provided detailed instruction sheets for the appropriate use of the pertinent vaginal device in the clinic [Supplementary Appendix 1 and 2]. All participants used the self-collected technique in the office bathroom immediately before the study speculum-based specimen collection. They placed the used vaginal devices into the original packaging and then directly into a sterile bag which we transported to the laboratory. There, they were stored dry at room temperature until DNA isolation. The speculum-based technique collected the standard of care cervical transformation zone sampling for cervical cancer screening before the scheduled clinical appointment. In other words, the self- and speculum-collected study specimens were completed before the colposcopy or routine co-testing (cytology and HPV) for primary screening scheduled that day.

### 2.3 DNA Isolation and HPV detection

We used a target-amplification polymerase chain reaction (PCR) HPV genotype platform. The HPV detection system (HPV PCR MassArray Assay) detects and identifies 18 high-risk HPV types, of which we measured the 15 high-risk HPV subtypes (16, 18, 31, 33, 35, 39, 45, 51, 52, 56, 58, 59, 66, 68 and 73) associated with cervical cancer. It includes interrogating human GAPDH as a control for sample DNA quality and assay validity for each HPV type (Brouwer et al., 2022; Ojesina et al., 2014). The assay employs type-specific, multiplex, competitive PCR to amplify the E6 region of HPV, followed by a probe-specific single base extension to discriminate between naturally occurring HPV present in the sample and the synthetic competitors included in the reaction. Matrix-assisted laser desorption/ionization-time of flight mass spectroscopy allows the separation of products on a matrix-loaded silicon chip array. Samples were run in quadruplicate with appropriate positive and negative controls. Both self- and speculum-directed samples remained at room temperature until DNA isolation [Supplementary Appendix 3].

The University of Michigan IRBMED approved this study (HUM00163299).

### 2.4 Statistical analysis

We measured the *prevalence and 95 % confidence interval* (95 %CI) of sixteen HPV sets: one pooled overall and each of the 15 HPV genotypes for the total population (colposcopy and primary care screening cohorts) from the self and speculum technique. We measured the agreement between self and speculum techniques for HPV detection by the positive percent agreement (PPA) and negative percent agreement (NPA) with 95 % CI (US FDA, 2007). In addition, we measured the concordance with *Prevalence-adjusted-kappa*, adjusting for the possibility of a low prevalence of the HPV type (Byrt et al., 1993). We used *Exact McNemar's test* to estimate any systematic differences in the marginal detection probability.

We powered the study on the proportion of discordant HPV test results in the 2 × 2 tables using McNemar's test for the entire population. We assumed the proportion of discordant results by collection technique would be less than 20 %; thereby, with 95 persons in each cohort (HPV positive/HPV negative), we have 90 % power at a 5 % significance level to see a difference of 10 % in the marginal probabilities.

We define prevalence-adjusted kappa thresholds as 0–0.2 as poor agreement, 0.21–0.4 as fair agreement, 0.41–0.6 as moderate agreement, 0.61–0.8 as substantial agreement, and 0.81–1.0 as almost perfect agreement(Landis and Koch, 1977). We define equivalent agreement between self and speculum-based collection techniques for those with a histologic biopsy report as a PPA for HPV 16 of 95 % with the lower bound of the 95 % CI greater than 81 %, the threshold for nearly perfect agreement, as defined in other studies (Arbyn et al., 2018a; Latsuzbaia et al., 2022). Once we meet this prerequisite, we define equivalent agreement in technique and vaginal device detection for remaining HPV types using Kappa and McNemar's statistics.

We measured the absolute and relative sensitivity and specificity of the HPV test for CIN 2+ disease for both the self and speculum techniques ((self compared to speculum technique) and 95 %CI) to provide a direct comparison between the two sampling techniques (Latsuzbaia et al., 2023a).

We used SAS 9.4 for all statistics (SAS 9.4, Cary, NC).

## 3 Results

### 3.1 Population

Of all invitees, 39 % agreed to sign the consent before their scheduled colposcopy appointment and 29 % before their primary care screening appointment. Of all consented, 93 % attended colposcopy, and 94 % attended primary care screening.

The average age of the 30–65-year-old enrollees was 45.9 (SD 10.5) years for those attending colposcopy and 46.2 (11.0) years for those attending primary care screening (Table 1). There were no differences in race/ethnicity distribution, reproductive status, pregnancy history, and the use of immunosuppressive medications by study cohort. The age at HPV vaccine initiation was significantly older among the colposcopy cohort compared to the primary care screening cohort (30.7 (16.5) vs. 20.3 (3.7), *p* = 0.052). There were no differences in the type of HPV vaccine received or completion by the study cohort. The incidence of past positive HPV tests, colposcopy exams, and excisional procedures occurred in the colposcopy cohort significantly more often than in the screening cohort (76 % vs. 8 %, 64 % vs. 16 %, 28 % vs. 2 %, *p* < 0.001, respectively).

**Table 1:** Descriptors of the US-based trial comparing Human Papillomavirusƚ collection devices by high and average-risk populations, 2020-2022.

	Colposcopy *N* = 97	Primary Care Screening *N* = 96
	N (%)	N (%)
**Age (mean, SD)**	45.9 (10.5)	46.2 (11.0)
**Race**		
Asian	3 (3)	2 (2)
Black/African American	10 (10)	5 (5)
MENAǂ	1 (1)	1 (1)
Multiracial	1 (1)	2 (2)
Multiracial/Hispanic	1 (1)	0
White	77 (79)	80 (83)
White/Hispanic	0	1 (1)
Hispanic, other races not described	1 (1)	4 (4)
Race not described	3 (3)	1 (1)
**Reproductive Status**		
Reproductive	60 (62)	58 (60)
Menopausal	37 (38)	38 (40)
**Pregnancy Status**		
Gravidity (mean, SD)	2.4 (2.1)	2.1 (1.5)
Parity (mean, SD)	1.6 (1.3)	1.6 (1.3)
**Age at first HPVƚ vaccination (mean, SD)**⁎	30.7 (16.5)	20.3 (3.7)
**HPVƚ vaccination initiated**		
Gardasil 4	5 (5)	8 (8)
Gardasil 9	7 (7)	3 (3)
Both	1 (1)	0
None	84 (87)	85 (89)
**HPVƚ vaccination completed**		
Yes	7 (7)	8 (8)
No	6 (6)	3 (3)
**Immunosuppressive medication currently**		
Yes	8 (8)	2 (2)
No	89 (92)	94 (98)
**Positive HPVƚ test in the past five years**		
Yes	74 (76)	8 (8)
No	23 (24)	88 (92)
**Colposcopy visits in the past**		
Yes	62 (64)	15 (16)
No	35 (36)	81 (84)
**LEEP§ procedure in the past**		
Yes	27 (28)	2 (2)
No	70 (72)	94 (98)

ƚHPV means human papillomavirus.

§ Loop electrosurgical excision procedure (LEEP).

ǂ Mid-Eastern-North African descent.

⁎ p = 0.05, by Chi-square testing.

### 3.2 HPV prevalence

Among the colposcopy cohort, the prevalence of any HPV was 66 % (95 % CI: 56–75 %), and in the primary care screening cohort, 14 % (8 %, 22 %) (Table 2]) HPV 16, 68, and 39 were the most commonly detected genotypes from both cohorts and by both collection techniques (Table 2). The majority of HPV-infected persons had one genotype (53/193 (27 %)), with HPV 16 being the most common regardless of collection technique (**Supplementary Table 1**). Of those with two genotypes (18/193 (9 %)), HPV 16 was the most commonly paired, and only 6/193 (3 %) had three or more HPV genotypes (**Supplementary Table 2**). HPV 73 is not included in commercial HPV testing platforms but was detected at least as often as HPV 31, 33, 35, 51, 56, 58, and 66 in self and speculum-based sampling.

**Table 2:** Prevalence of Human Papillomavirus positivity by genotype, cohort, and collection technique among the high and average risk US population, 2020–2022.

Either self or speculum technique	Population					
	Total (*N* = 193)		Colposcopy Cohort (N = 97)		Primary Care Screening Cohort (N = 96)	
	Positive	Percent (95 % CI)	Positive	Percent (95 % CI)	Positive	Percent (95 % CI)
Overall HPV§	77	40 % (33 %, 47 %)	64	66 % (56 %, 75 %)	13	14 % (8 %, 22 %)
HPV 16	29	15% (11 %, 21 %)	23	24 % (16 %, 33 %)	6	6 % (3 %, 13 %)
HPV 68	13	7 % (4 %, 11 %)	11	11 % (6 %, 19 %)	2	2 % (0 %, 8 %)
HPV 39	11	6 % (3 %, 10 %)	9	9 % (5 %, 17 %)	2	2 % (0 %, 8 %)
HPV 18	10	5 % (3 %, 9 %)	9	9 % (5 %, 17 %)	1	1 % (0 %, 6 %)
HPV 45	8	4% (2 %, 8 %)	7	7 % (3 %, 14 %)	1	1 % (0 %, 6 %)
HPV 52	6	3 % (1 %, 7 %)	5	5 % (2 %, 12 %)	1	1 % (0 %, 6 %)
HPV 56	6	3 % (1 %, 7 %)	5	5 % (2 %, 12 %)	1	1 % (0 %, 6 %)
HPV 31	5	3 % (1 %, 6 %)	3	3 % (1 %, 9 %)	2	2 % (0 %, 8 %)
HPV 58	5	3 % (1 %, 6 %)	5	5 % (2 %, 12 %)	0	
HPV 59	5	3 % (1 %, 6 %)	5	5 % (2 %, 12 %)	0	
HPV 66	5	3 % (1 %, 6 %)	5	5 % (2 %, 12 %)	0	
HPV 73	5	3 % (1 %, 6 %)	5	5 % (2 %, 12 %)	0	
HPV 35	4	2 % (1 %, 5 %)	3	3 % (1 %, 9 %)	1	1 % (0 %, 6 %)
HPV 51	3	2 % (0 %, 5 %)	3	3 % (1 %, 9 %)	0	
HPV 33	2	1 % (0 %, 4 %)	2	2 % (0 %, 8 %)	0	

	**Total (N = 193)**		**Colposcopy Cohort (*N* = 97)**		**Primary Care Screening Cohort (N = 96)**	
**Self-collection**	Positive	Percent (95 % CI)	Positive	Percent (95 % CI)	Positive	Percent (95 % CI)
Overall HPV	70	36 % (30 %, 43 %)	58	60 % (50 %, 69 %)	12	13 % (7 %, 21 %)
HPV 16	27	14 % (10 %, 20 %)	22	23 % (15 %, 32 %)	5	5% (2 %, 12 %)
HPV 68	11	6 % (3 %, 10 %)	9	9 % (5 %, 17 %)	2	2 % (0 %, 8 %)
HPV 39	9	5% (2 %, 9 %)	7	7 % (3 %, 14 %)	2	2% (0 %, 8 %)
HPV 45	7	4% (2 %, 7 %)	6	6 % (3 %, 13 %)	1	1 % (0 %, 6 %)
HPV 18	6	3 % (1 %, 7 %)	5	5% (2 %, 12 %)	1	1 % (0 %, 6 %)
HPV 52	6	3 % (1 %, 7 %)	5	5 % (2 %, 12 %)	1	1 % (0 %, 6 %)
HPV 31	5	3 % (1 %, 6 %)	3	3 % (1 %, 9 %)	2	2 % (0 %, 8 %)
HPV 59	5	3 % (1 %, 6 %)	5	5% (2 %, 12 %)	0	
HPV 35	4	2% (1 %, 5 %)	3	3 % (1 %, 9 %)	1	1 % (0 %, 6 %)
HPV 56	4	2% (1 %, 5 %)	3	3 % (1 %, 9 %)	1	1 % (0 %, 6 %)
HPV 58	4	2% (1 %, 5 %)	4	4% (1 %, 10 %)	0	
HPV 66	4	2% (1 %, 5 %)	4	4% (1 %, 10 %)	0	
HPV 73	4	2% (1 %, 5 %)	4	4% (1 %, 10 %)	0	
HPV 51	3	2% (0 %, 5 %)	3	3 % (1 %, 9 %)	0	
HPV 33	2	1 % (0 %, 4 %)	2	2 % (0 %, 8 %)	0	

	**Total (*N* = 193)**		**Colposcopy Cohort (N = 97)**		**Primary Care Screening Cohort (*N* = 96)**	
**Speculum collection**	Positive	Percent (95 % CI)	Positive	Percent (95 % CI)	Positive	Percent (95 % CI)
Overall HPV	71	37 % (30 %, 44 %)	61	63 % (53 %, 72 %)	10	10 % (6 %, 18 %)
HPV 16	28	15% (10 %, 20 %)	22	23 % (15 %, 32 %)	6	6 % (3 %, 13 %)
HPV 68	11	6 % (3 %, 10 %)	9	9 % (5 %, 17 %)	2	2% (0 %, 8 %)
HPV 39	10	5 % (3 %, 9 %)	8	8 % (4 %, 16 %)	2	2% (0 %, 8 %)
HPV 18	9	5% (2 %, 9 %)	9	9 % (5 %, 17 %)	0	
HPV 45	8	4% (2 %, 8 %)	7	7 % (3 %, 14 %)	1	1 % (0 %, 6 %)
HPV 52	6	3 % (1 %, 7 %)	5	5% (2 %, 12 %)	1	1 % (0 %, 6 %)
HPV 56	5	3 % (1 %, 6 %)	5	5 % (2 %, 12 %)	0	
HPV 59	5	3 % (1 %, 6 %)	5	5% (2 %, 12 %)	0	
HPV 66	5	3 % (1 %, 6 %)	5	5% (2 %, 12 %)	0	
HPV 35	4	2% (1 %, 5 %)	3	3 % (1 %, 9 %)	1	1 % (0 %, 6 %)
HPV 73	4	2% (1 %, 5 %)	4	4 % (1 %, 10 %)	0	
HPV 31	3	2% (0 %, 5 %)	3	3 % (1 %, 9 %)	0	
HPV 58	3	2% (0 %, 5 %)	3	3 % (1 %, 9 %)	0	
HPV 33	1	1 % (0 %, 3 %)	1	1 % (0 %, 6 %)	0	
HPV 51	1	1 % (0 %, 3 %)	1	1 % (0 %, 6 %)	0	

§HPV means human papillomavirus.

The same six genotypes are the most prevalent in both cohorts, albeit with different frequencies.

### 3.3 Agreement between self vs. speculum collection techniques

We have the power to detect a difference between self and speculum-based collection techniques because the discordant results for the entire population are less than 20 %, ranging from 0 % to 7 % (Table 3).

**Table 3:** Agreement between self and speculum techniques for Human Papillomavirus genotypes by high and average risk US population, 2020–2022.

Population	Self Positive/ Speculum Positive	Self Positive/ Speculum Negative	Self Negative/ Speculum Positive	Self Negative/ Speculum Negative	Positive Percent Agreement (95 % CI)	Negative Percent Agreement (95 % CI)	Prevalence Adjusted Kappa	Exact McNemar's p-value
							(95 % CI)	
**Colposcopy Cohort (N = 97)**								
Overall HPV§	55	3	6	33	90.1	91.7	0.81	0.51
					(82.7, 97.6)	(82.6, 100)	(0.70, 0.93)	
HPV 16	21	1	1	74	95.5	98.7	0.96	1
					(86.8, 100)	(96.1, 100)	(0.90, 1)	
HPV 18	5	0	4	88	55.6	100	0.92	0.13
					(23.1, 88.0)	(100,100)	(0.84, 1.00)	
HPV 31	3	0	0	94	100	100	–	–
					(100, 100)	(100, 100)		
HPV 33	1	1	0	95	100	99	0.98	1
					(100, 100)	(96.9, 100)	(0.94, 1)	
HPV 35	3	0	0	94	100	100	–	–
					(100, 100)	(100, 100)		
HPV 39	6	1	2	88	75	98.9	0.94	1
					(45.0, 100)	(96.7, 100)	(0.87, 1)	
HPV 45	6	0	1	90	85.7	100	0.98	1
					(59.8, 100)	(100, 100)	(0.94, 1)	
HPV 51	1	2	0	94	100	97.9	0.96	0.5
					(100, 100)	(95.1, 100)	(0.90, 1)	
HPV 52	5	0	0	92	100	100	–	–
					(100, 100)	(100, 100)		
HPV 56	3	0	2	92	60	100	0.96	0.5
					(17.1, 100)	(100, 100)	(0.90, 1)	
HPV 58	2	2	1	92	66.7	97.9	0.94	1
					(13.3, 100)	(95.0, 100)	(0.87, 1)	
HPV 59	5	0	0	92	100	100	–	–
					(100, 100)	(100, 100)		
HPV 66	4	0	1	92	80	100	0.98	1
					(44.9, 100)	(100, 100)	(0.94, 1)	
HPV 68	7	2	2	86	77.8	97.7	0.92	1
					(50.6, 100)	(94.6, 100)	(0.84, 1)	
HPV 73	3	1	1	92	75	98.9	0.96	1
					(32.6, 100)	(96.8, 100)	(0.90, 1)	
**Primary Care Screening Cohort (N = 96)**								
Overall HPV	9	3	1	83	90	96.5	0.92	0.63
					(71.4, 100)	(92.6, 100)	(0.84, 1.00)	
HPV 16	5	0	1	90	83.3	100	0.98	1
					(53.5, 100)	(100, 100)	(0.94, 1.00)	
HPV 18	0	1	0	95		99	–	–
						(96.9, 100)		
HPV 31	0	2	0	94		97.9	–	–
						(95.1, 100)		
HPV 33	0	0	0	96		100	–	–
						(100, 100)		
HPV 35	1	0	0	95	100	100	–	–
					(100, 100)	(100, 100)		
HPV 39	2	0	0	94	100	100	–	–
					(100, 100)	(100, 100)		
HPV 45	1	0	0	95	100	100	–	–
					(100, 100)	(100, 100)		
HPV 51	0	0	0	96		100	–	–
						(100, 100)		
HPV 52	1	0	0	95	100	100	–	–
					(100, 100)	(100, 100)		
HPV 56	0	1	0	95		99	–	–
						(96.9, 100)		
HPV 58	0	0	0	96		100	–	–
						(100, 100)		
HPV 59	0	0	0	96		100	–	–
						(100, 100)		
HPV 66	0	0	0	96		100	–	–
						(100, 100)		
HPV 68	2	0	0	94	100	100	–	–
					(100, 100)	(100, 100)		
HPV 73	0	0	0	96		100	–	–
						(100, 100)		
**Entire Cohort (N = 193)**								
Overall HPV	64	6	7	116	90.1	95.1	0.87	1
					(83.2, 97.1)	(91.2, 98.9)	(0.80, 0.94)	
HPV 16	26	1	2	164	92.9	99.4	0.97	1
					(83.3, 100)	(98.2, 100)	(0.93, 1)	
HPV 18	5	1	4	183	55.6	99.5	0.95	0.38
					(23.1, 88.0)	(98.4, 100)	(0.90, 0.99)	
HPV 31	3	2	0	188	100	98.9	0.98	0.5
					(100, 100)	(97.5, 100)	(0.95, 1)	
HPV 33	1	1	0	191	100	99.5	0.99	1
					(100, 100)	(98.5, 100)	(0.97, 1)	
HPV 35	4	0	0	189	100	100	–	–
					(100, 100)	(100, 100)		
HPV 39	8	1	2	182	80	99.5	0.97	1
					(55.2, 100)	(98.4, 100)	(0.93, 1)	
HPV 45	7	0	1	185	87.5	100	0.99	1
					(64.6, 100)	(100, 100)	(0.97, 1)	
HPV 51	1	2	0	190	100	99	0.98	0.5
					(100, 100)	(97.5, 100)	(0.95, 1)	
HPV 52	6	0	0	187	100	100	–	–
					(100, 100)	(100, 100)		
HPV 56	3	1	2	187	60	99.5	0.97	1
					(17.1, 100)	(98.4, 100)	(0.93, 1)	
HPV 58	2	2	1	188	66.7	99	0.97	1
					(13.3, 100)	(97.5, 100)	(0.93, 1)	
HPV 59	5	0	0	188	100	100	–	–
					(100, 100)	(100, 100)		
HPV 66	4	0	1	188	80	100	0.99	1
					(44.9, 100)	(100, 100)	(0.97, 1)	
HPV 68	9	2	2	180	81.8	99	0.96	1
					(59.0, 100)	(97.4, 100)	(0.92, 1)	
HPV 73	3	1	1	188	75	99.5	0.98	1
					(32.6, 100)	(98.4, 100)	(0.95, 1)	

§HPV means human papillomavirus.

### 3.4 Colposcopy cohort

The HPV 16-PPA was 96 % (87 %, 100 %) for the self vs. speculum collection technique among the colposcopy cohort, indicating almost perfect agreement (*prevalence adjusted kappa* 0.96 (0.90, 1.00)), meeting our pre-defined level of agreement for further comparisons. Likewise, it has non-significant discordant margins (McNemar's *p*-value =1) (Table 3). Moreover, the NPA for HPV 16 was 98.7 % (96.1 %, 100 %) for comparing self vs. speculum collection techniques.

Further, between collection techniques, almost perfect HPV-type detection agreement occurred with prevalence-adjusted kappa values above the 0.81 threshold and non-significant McNemar's discordant margins for each HPV genotype comparison between the self vs. speculum techniques. Even with the lowest PPA of 56 % (23 %, 88 %), occurring for HPV 18, the kappa agreement (0.92 (0.84,1.00)) between self and speculum was almost perfect with the lower bound of the 95 % CI above 0.81, and non-significant McNemar's *p*-values for self vs. speculum collection. Six HPV genotypes had 100 % PPA, and eight had 100 % NPA.

### 3.5 Primary care screening cohort

The *prevalence-adjusted kappa comparisons* were only possible for HPV 16 and all pooled HPV types due to a much lower prevalence of HPV infection than the colposcopy cohort (Table 3). Both kappa agreements for HPV 16 and overall HPV were above the 0.81 threshold for the lower bound of the 95 % CI for self vs. speculum collection, with non-significant McNemar's p-values for discordant margins, signifying almost perfect agreement (HPV 16 at 0.98 (0.94, 1.00) and overall HPV at 0.92 (0.84, 1.00), respectively).

Moreover, for the primary care cohort, the PPA values were excellent: the PPA was 90 % (71 %, 100 %) for any HPV type, 83 % (54 %, 100 %) for HPV 16, and 100 % for each type detected. In addition, the NPA was 97 % (93 %, 100 %) for any HPV type and 100 % for HPV 16, with 100 % agreement for all other types except HPV 18 (99 % (97 %, 100 %) and HPV 31 (98 % (95 %, 100 %).

### 3.6 Diseased vs. non-diseased agreement

For overall HPV positivity and individual HPV genotypes among people with CIN 2+ disease detected, an agreement between self- and speculum-based sampling was perfect (*prevalence-adjusted kappa is 1.00*) **(**Table 4). Of the colposcopy cohort, 16 had CIN2+ disease; of the primary care cohort, three required colposcopy, and one had CIN2+ (17 women with CIN2+). An almost perfect agreement exists for HPV 45, where kappa was 0.88 (0.66, 1.00), with a non-significant McNemar's at 1.0. Among the people with less than CIN 2 disease detected, kappa scores remained above the 0.81 threshold for almost-perfect agreement for all individual types.

**Table 4:** Human Papillomavirus genotype agreement by screening technique based on those with a clinical threshold of Cervical Intraepithelial Neoplasia Grade 2 or worse, for the US population who had a histologic endpoint, 2020–2022.

CIN2 + ǂ	HPVƚ type	+self/ +speculum	+self/ -speculum	-self/ +speculum	-self/ -speculum	Positive Percent Agreement (95 % CI)	Negative Percent agreement (95 % CI)	Prevalence Adjusted Kappa (95 % CI)	Exact McNemar's p-value
***n* = 17**	all	14	0	0	3	1.00 (1.00, 1.00)	1.00 (1.00, 1.00)	1.00 (1.00, 1.00)	
	16	7	0	0	10	1.00 (1.00, 1.00)	1.00 (1.00, 1.00)	1.00 (1.00, 1.00)	
	18	1	0	0	16	1.00 (1.00, 1.00)	1.00 (1.00, 1.00)	1.00 (1.00, 1.00)	
	31	0	0	0	17		1.00 (1.00, 1.00)		
	33	0	0	0	17		1.00 (1.00, 1.00)		
	35	1	0	0	16	1.00 (1.00, 1.00)	1.00 (1.00, 1.00)	1.00 (1.00, 1.00)	
	39	1	0	0	16	1.00(1.00, 1.00)	1.00 (1.00, 1.00)	1.00 (1.00, 1.00)	
	45	2	0	1	14	0.67 (0.13, 1.00)	1.00 (1.00, 1.00)	0.88 (0.66, 1.00)	1.00
	51	0	1	0	16		0.94 (0.83, 1.00)		
	52	0	0	0	17		1.00 (1.00, 1.00)		
	56	0	0	0	17		1.00 (1.00, 1.00)		
	58	1	0	0	16	1.00 (1.00, 1.00)	1.00 (1.00, 1.00)	1.00 (1.00, 1.00)	
	59	1	0	0	16	(1.00, 1.00)	1.00 (1.00, 1.00)	1.00 (1.00, 1.00)	
	66	1	0	0	16	1.00 (1.00, 1.00)	1.00 (1.00, 1.00)	1.00 (1.00, 1.00)	
	68	1	0	0	16	1.00 (1.00, 1.00)	1.00 (1.00, 1.00)	1.00 (1.00, 1.00)	
	73	0	0	0	17		1.00 (1.00, 1.00)		
<CIN 2	all	44	4	6	28	0.88 (0.79, 0.97)	0.88 (0.76, 0.99)	0.76 (0.61, 0.90)	0.75
*n* = 82	16	14	1	1	66	0.93 (0.81, 1.00)	0.99 (0.96, 1.00)	0.95 (0.88, 1.00)	1.00
	18	4	0	4	74	0.50 (0.15, 0.85)	1.00 (1.00, 1.00)	0.90 (0.81, 1.00)	0.13
	31	3	0	0	79	1.00 (1.00, 1.00)	1.00 (1.00, 1.00)	1.00 (1.00, 1.00)	
	33	1	1	0	80	1.00 (1.00, 1.00)	0.99 (0.96, 1.00)	0.98 (0.93, 1.00)	1.00
	35	3	0	0	79	1.00 (1.00, 1.00)	1.00 (1.00, 1.00)	1.00 (1.00, 1.00)	
	39	7	1	2	72	0.78 (0.51, 1.00)	0.99 (0.96, 1.00)	0.93 (0.85, 1.00)	1.00
	45	5	0	0	77	1.00 (1.00, 1.00)	1.00 (1.00, 1.00)	1.00 (1.00, 1.00)	
	51	1	1	0	80	1.00 (1.00, 1.00)	0.99 (0.96, 1.00)	0.98 (0.93, 1.00)	1.00
	52	6	0	0	76	1.00 (1.00, 1.00)	1.00 (1.00, 1.00)	1.00 (1.00, 1.00)	
	56	3	0	2	77	0.60 (0.17, 1.00)	1.00 (1.00, 1.00)	0.95 (0.88, 1.00)	0.50
	58	1	2	1	78	0.50 (0, 1.00)	0.98 (0.94, 1.00)	0.93 (0.85, 1.00)	1.00
	59	4	0	0	78	1.00 (1.00, 1.00)	1.00 (1.00, 1.00)	1.00 (1.00, 1.00)	
	66	3	0	1	78	0.75 (0.33, 1.00)	1.00 (1.00, 1.00)	0.98 (0.93, 1.00)	1.00
	68	6	2	2	72	0.75 (0.45, 1.00)	0.97 (0.94, 1.00)	0.90 (0.81, 1.00)	1.00
	73	3	1	1	77	0.75 (0.33, 1.00)	0.99 (0.96, 1.00)	0.95 (0.88, 1.00)	1.00

ǂCIN2+ means cervical intraepithelial neoplasia grade 2 or worse.

ƚHPV means human papillomavirus.

### 3.7 Test performance characteristics against the clinical CIN 2+ disease threshold

We compared the sensitivity and specificity of self- and speculum-based techniques using comparative ratios for people with CIN 2+ detection to those less than CIN 2.

Table 4 indicates that among the colposcopy and primary care screening groups combined, 17 cases of CIN2+ were detected. Of these, 14 cases were HPV positive by both self-sampling and speculum sampling, highlighting the clinical relevance of these collection methods in identifying high-grade lesions.

Table 5 shows that for the pooled overall HPV test, the sensitivity and specificity ratios are not different at 1.00 (0.29, 3.45) and 1.06 (0.61, 1.83), respectively.

**Table 5:** Accuracy of Human Papillomavirus detection for Cervical Intraepithelial Neoplasia Grade 2 or worse by collection technique among US women with a histologic endpoint, 2020–2022.

HPV ƚ genotype	self test x biopsy result				speculum test x biopsy result				Sensitivity (95 % CI)		Specificity (95 % CI)		Sensitivity Ratio (95 % CI)	Specificity Ratio (95 % CI)
	True Positive	False Positive	False Negative	True Negative	True Positive	False Positive	False Negative	True Negative	self collect	speculum collect	self collect	speculum collect	self/ speculum	self/ speculum
Overall	14	48	3	34	14	50	3	32	0.82 (0.64, 1.00)	0.82 (0.64, 1.00)	0.41 (0.31, 0.52)	0.39 (0.28, 0.50)	1.00 (0.37, 2.72)	1.06 (0.60, 1.88)
HPV 16	7	15	10	67	7	15	10	67	0.41 (0.18, 0.65)	0.41 (0.18, 0.65)	0.82 (0.73, 0.90)	0.82 (0.73, 0.90)	1.00 (0.29, 3.47)	1.00 (0.63, 1.58)
HPV 18	1	4	16	78	1	8	16	74	0.06 (0, 0.17)	0.06 (0, 0.17)	0.95 (0.90, 1.00)	0.90 (0.84, 0.97)	1.00 (0.06, 17.33)	1.05 (0.68,1.64)
HPV 31	0	3	17	79	0	3	17	79	0 (0,0)	0 (0, 0)	0.96 (0.92, 1.00)	0.96 (0.92, 1.00)	–	1.00 (0.65, 1.55)
HPV 33	0	2	17	80	0	1	17	81	0 (0, 0)	0 (0, 0)	0.98 (0.94, 1.00)	0.99 (0.96, 1.00)	–	0.99 (0.64, 1.53)
HPV 35	1	3	16	79	1	3	16	79	0.06 (0, 0.17)	0.06 (0, 0.17)	0.96 (0.92, 1.00)	0.96 (0.92, 1.00)	1.00 (0.06, 17.33)	1.00 (0.65, 1.55)
HPV 39	1	8	16	74	1	9	16	73	0.06 (0, 0.17)	0.06 (0, 0.17)	0.90 (0.84, 0.97)	0.89 (0.82, 0.96)	1.00 (0.06, 17.33)	1.01 (0.65, 1.58)
HPV 45	2	5	15	77	3	5	14	77	0.12 (0, 0.27)	0.18 (0, 0.36)	0.94 (0.89, 0.99)	0.94 (0.89, 0.99)	0.67 (0.10, 4.51)	1.00 (0.64, 1.55)
HPV 51	1	2	16	80	0	1	17	81	0.06 (0, 0.17)	0 (0, 0)	0.98 (0.94, 1.00)	0.99 (0.96, 1.00)	–	0.99 (0.64, 1.53)
HPV 52	0	6	17	76	0	6	17	76	0 (0, 0)	0 (0, 0)	0.93 (0.87, 0.98)	0.93 (0.87, 0.98)	–	1.00 (0.64, 1.55)
HPV 56	0	3	17	79	0	5	17	77	0 (0, 0)	0 (0, 0)	0.96 (0.92, 1.00)	0.94 (0.89, 0.99)	–	1.03 (0.66, 1.59)
HPV 58	1	3	16	79	1	2	16	80	0.06 (0, 0.17)	0.06 (0, 0.17)	0.96 (0.92, 1.00)	0.98 (0.94, 1.00)	1.00 (0.06, 17.33)	0.99 (0.64, 1.53)
HPV 59	1	4	16	78	1	4	16	78	0.06 (0, 0.17)	0.06 (0, 0.17)	0.95 (0.90, 1)	0.95 (0.90, 1)	1.00 (0.06, 17.33)	1.00 (0.65, 1.55)
HPV 66	1	3	16	79	1	4	16	78	0.06 (0, 0.17)	0.06 (0, 0.17)	0.96 (0.92, 1.00)	0.95 (0.90, 1)	1.00 (0.06, 17.33)	1.01 (0.65, 1.57)
HPV 68	1	8	16	74	1	8	16	74	0.06 (0, 0.17)	0.06 (0, 0.17)	0.90 (0.84, 0.97)	0.90 (0.84, 0.97)	1.00 (0.06, 17.33)	1.00 (0.64, 1.56)
HPV 73	0	4	17	78	0	4	17	78	0 (0, 0)	0 (0, 0)	0.95 (0.90, 1)	0.95 (0.90, 1)	–	1.00 (0.65, 1.55)

ƚ HPV means human papillomavirus.

The false positive ratios (FPR) varied between 0.50 and 2.0 across all genotypes with broad confidence intervals (Supplementary Table 3); eight genotypes had FPRs at 1.0. Likewise, the false negative ratios (FNR) anchor at or near 1.00 for each genotype, except for HPV 18, 56, and 66.

## 4 Discussion

Our work is the first US-based prospective study powered to detect differences between HPV genotype detection by self-collection vs. speculum-based collection in a well-screened population. Our work recruited participants in all ranges of disease states from general and disease specialty clinics across all ages 30–65 and all races/ethnicities presenting for care, minimizing any spectrum bias. In addition, we used a research-based assay using targeted amplification, the only assay type recommended for primary HPV testing in the literature and the only kind of assay FDA-approved for primary HPV testing (Arbyn et al., 2022). We did not use a commercially available assay because we were interested in individually non-aggregated HPV genotypes. We have minimized all biases pertinent to diagnostic accuracy reporting.

Our work indicates vaginal devices do not differ from the speculum-based collection for primary HPV testing for cervical cancer screening as seen in other global work (Arbyn et al., 2022; Arbyn et al., 2018b; Fleider et al., 2023; Latsuzbaia et al., 2022). Our high prevalence-adjusted kappa values with tight confidence intervals indicate superb concordance for overall HPV and each genotype, including HPV 73, not included in the commercial tests for diseased and non-diseased populations.

Our population mirrors other globally reported populations. Our primary care general population HPV prevalence is 14 % for people 30–65 years, 10 % positive for a single type, 3.1 % for multiple types, and 6 % HPV 16 positive, agreeing with other general population prevalence rates (Bruni et al., 2010; Han et al., 2021). Our HPV prevalence rates in the colposcopy clinic were similar to other reports, where rates ranged from 57 %–87 % (Berza et al., 2024; Cadman et al., 2021; Latsuzbaia et al., 2022; Martinelli et al., 2019).

According to FDA guidance, the PPA and NPA with 95 % CI must be a reported measure of accuracy. In our study, the PPA, NPA, and prevalence-adjusted kappa scores were almost perfect, with narrow confidence intervals for all HPV genotypes agreeing with that found in other work (Martinelli et al., 2023). Moreover, our kappa values were superior, with tighter confidence intervals than those published with some commercial target amplification assays (Arbyn et al., 2022; Latsuzbaia et al., 2023b; Latsuzbaia et al., 2022).

When the reference standard, the presence of CIN2+ disease, independent of the test performance and based on independently reviewed histology, is applied to PPA calculations, we show a perfect 1.0 PPA for all but one HPV genotype, with very tight confidence intervals. Likewise, our NPA calculations anchor at 1.0 with tight confidence intervals. Using the histologic endpoint of CIN2+, the absolute and relative sensitivity, and specificity of HPV detection to predict CIN2+ allows us to conclude that self and speculum-based collection techniques are equivalent. Our sensitivity and specificity ratios indicating equivalent collection techniques matched those found in a large meta-analysis where the HPV detection platform was PCR-based, similar to ours (Arbyn et al., 2018b).

### 4.1 Strengths and limitations

This study is novel, being the first to study a US-based population to investigate HPV genotype concordance between self-collection and speculum-based collection. The agreement kappa values are nearly perfect with very tight confidence intervals, indicating that HPV detection among people with CIN 2+ is equivalent by self and speculum-based techniques. Indeed, the positive and negative percent agreements are likewise nearly perfect, with very tight confidence intervals. This level of agreement meets the regulatory framework set by the FDA for clinical validation of self- vs. speculum-based HPV collection for cervical cancer screening.

Our study's limitations include 99 participants with histologic outcomes from the screening process, restricted to those who underwent colposcopy. One framework recommends enrolling 118 people with CIN2+ disease to calculate sensitivity and specificity ratios with narrow confidence intervals. However, our US-based study results do not contradict any evaluation study to date (Arbyn et al., 2018a).

## 5 Conclusions

Our findings confirm that primary HPV screening using self-collection techniques is equivalent to speculum-based collection in detecting HPV among women aged 30–65. The almost perfect agreement in kappa values for all detected HPV genotypes, including those not typically included in commercial tests like HPV 73, underscores the reliability of self-collection in varied clinical settings. This equivalence holds true across both diseased {high-grade lesions) and non-diseased populations, suggesting that self-collection could be a viable alternative to speculum-based collection, offering increased screening accessibility and comfort.

Furthermore, the detection rates of CIN2+ among our study participants provide critical insights into the clinical relevance of these collection methods. The ability of both collection techniques to effectively identify high-grade lesions supports their use in routine cervical cancer screening protocols. Our study also aligns with global data on HPV prevalence and genotype distribution, reinforcing the validity of our findings within broader epidemiological contexts.

Considering our comprehensive analysis and the rigorous comparison of self-collection and speculum-based methods, we advocate for integrating self-collection into standard HPV screening practices, especially in settings where traditional methods are challenging to implement. This recommendation is supported by our study's alignment with FDA guidelines on diagnostic accuracy and the proven equivalence in sensitivity and specificity between the two collection techniques.

## Funding

**UM1TR004404** – MICHR funding.

**P30CA046592** – NCI Rogel Cancer Center funding.

## Submission declaration

This work has not been previously published. It is not under consideration for publication elsewhere. All authors approve of the article.

## Authorship contribution

All have participated in the revisions and final editing of all manuscript drafts and in interpreting the data. As this is a major article for US practice patterns, all authors have been critically involved in its writing and revisions. All authors approve the final version resubmitted herein. All listed authors meet the criteria for authorship. All authors are part of the study group MISSH, the group authorship name.

## Disclosure of interests

Conflict of interest statements are filed as the ICJME forms for each author. There are no conflicts of interest.

## CRediT authorship contribution statement

**Alisa P. Young:** Writing – review & editing, Validation, Project administration, Investigation, Data curation. **Mutiya Olorunfemi:** Writing – review & editing, Validation, Investigation. **Leigh Morrison:** Writing – review & editing, Validation, Investigation, Conceptualization. **Scott A. Kelley:** Writing – review & editing, Validation, Data curation, Conceptualization. **Anna Laurie:** Writing – review & editing, Validation, Methodology. **Anna McEvoy:** Writing – review & editing, Validation, Methodology, Conceptualization. **Jill Schneiderhan:** Writing – review & editing, Validation, Investigation, Data curation. **Julie Prussack:** Writing – review & editing, Validation, Investigation. **Marie Claire O'Dwyer:** Writing – review & editing, Validation, Investigation, Data curation. **Pamela Rockwell:** Writing – review & editing, Validation, Investigation, Conceptualization. **Philip Zazove:** Writing – review & editing, Validation, Investigation, Conceptualization. **Jonathan Gabison:** Writing – review & editing, Validation, Investigation, Data curation. **Jane Chargot:** Writing – review & editing, Validation, Methodology, Data curation. **Kristina Gallagher:** Writing – review & editing, Validation, Methodology, Data curation. **Ananda Sen:** Writing – review & editing, Validation, Supervision, Methodology, Investigation, Formal analysis, Conceptualization. **Dongru Chen:** Writing – review & editing, Validation, Methodology, Formal analysis. **Elizabeth A. Haro:** Writing – review & editing, Validation, Supervision, Project administration, Methodology, Investigation. **Emma A. Butcher:** Writing – review & editing, Supervision, Project administration, Investigation, Data curation. **Martha L. Alves:** Writing – review & editing, Validation, Supervision, Project administration, Methodology, Investigation, Data curation. **Christelle El Khoury:** Writing – review & editing, Validation, Project administration, Methodology, Investigation, Data curation. **Melinda L. Dendrinos:** Writing – review & editing, Investigation, Conceptualization. **Nicole Brashear:** Writing – review & editing, Validation, Investigation. **Roger Smith:** Writing – review & editing, Validation, Investigation. **Richard W. Lieberman:** Writing – review & editing, Investigation, Conceptualization. **Natalie Saunders:** Writing – review & editing, Validation, Investigation, Data curation. **Elizabeth Campbell:** Writing – review & editing, Validation, Investigation. **Heather M. Walline:** Writing – review & editing, Validation, Resources, Methodology, Formal analysis. **Diane M. Harper:** Writing – review & editing, Writing – original draft, Validation, Supervision, Resources, Project administration, Methodology, Investigation, Funding acquisition, Data curation, Conceptualization.

## Declaration of competing interest

The authors declare that they have no known competing financial interests or personal relationships that could have appeared to influence the work reported in this paper.

## Data Availability

The authors do not have permission to share data.

## Appendix A Supplementary data

Supplementary material 1 Image 1

Supplementary material 2 Image 2

Supplementary material 3 Image 3

Supplementary material 4 Image 4
