# Supplementary material for: Cervical cancer screening: Impact of collection technique on human papillomavirus detection and genotyping
Source: Prev Med Rep. 2025 Jan 17;50:102971. doi: 10.1016/j.pmedr.2025.102971 (PMC11791345; doi:10.1016/j.pmedr.2025.102971)
Supplement: Supplementary file 1 — Supplementary material 1 [file mmc1.docx]

Supplementary Appendix. Deoxyribonucleic Acid (DNA) Isolation from US high and average-risk women from clinician and self-collected devices, 2020-2022.

Sample Preparation:

- If the sample is in a Thin Prep container, shake vigorously for 5 seconds and pour 10mL (milliliters) of the sample into a 15mL conical tube; proceed to step 1.
- If a swab or brush from a self-sample, add 10mL of Thin Prep solution into a 15mL conical tube. Place the swab or brush into the tube and allow to sit for at least 10 mins (minutes) at room temperature (RT). Longer is fine—vortex tube for 20-30secs. Dispose of swab or brush & proceed to step 1.

1. Centrifuge Thin Prep samples (in 15mL conical tubes) for 5 minutes at 2000rcf (relative centrifugal force) to pellet the cells.

2. Discard the supernatant by pipetting or pouring, leaving the pellet undisturbed.

3. Re-suspend the pellet in 1mL Cell Lysis Solution and mix by inverting. Incubate for 15 minutes at RT.

4. Add 10µL Proteinase K and vortex for 10 seconds. Incubate for 10 minutes at RT.

5. Add 340µL Protein Precipitation Solution and vortex for 10 seconds. Incubate for 10 minutes on ice.

6. Centrifuge for 10 minutes at 2000rcf. (If pellet is not tight, incubate on ice for 5 additional minutes and then repeat centrifugation). During centrifugation, pipette 1mL 100% Isopropanol and 2µL Glycogen into a clean 15mL conical tube.

7. Pipet supernatant into the tube containing Isopropanol and Glycogen. Mix by inverting.

8. Centrifuge for 5 minutes at 2000rcf and carefully discard supernatant.

9. Add 1mL 70% Ethanol to the pellet and mix by inverting to wash DNA pellet.

10. Centrifuge for 1 minute at 2000rcf at RT. Carefully pipet off the supernatant. Allow pellet to air dry at RT for 30 minutes (in the hood).

11. Add 50µL DNA Hydration Solution to the pellet and incubate at 4°C overnight to allow DNA to dissolve.
