# Supplementary material for: Cervical cancer screening: Impact of collection technique on human papillomavirus detection and genotyping
Source: Prev Med Rep. 2025 Jan 17;50:102971. doi: 10.1016/j.pmedr.2025.102971 (PMC11791345; doi:10.1016/j.pmedr.2025.102971)
Supplement: Supplementary file 2 — Supplementary material 2 [file mmc2.docx]

Supplementary Table 1. Prevalence of Human Papillomavirus genotypes by number of types per person from all US women enrolled by collection technique (N=193), 2020-2022

| **Among those with one genotype (N=53)** | | | |
| --- | --- | --- | --- |
| Self-collection (N=49) | | Speculum collection (N=48) | |
| **HPV ƚ types** | **N (%)** | **HPV types** | **N (%)** |
| HPV 16 | 17 (35%) | HPV 16 | 18 (38%) |
| HPV 18 | 4 (8%) | HPV 18 | 4 (8%) |
| HPV 58 | 4 (8%) | HPV 58 | 3 (6%) |
| HPV 41 | 3 (6%) | HPV 41 | 4 (8%) |
| HPV 66 | 3 (6%) | HPV 66 | 4 (8%) |
| HPV 52 | 3 (6%) | HPV 52 | 3 (6%) |
| HPV 68 | 3 (6%) | HPV 68 | 3 (6%) |
| HPV 51 | 3 (6%) | HPV 51 | 1 (2%) |
| HPV 35 | 2 (4%) | HPV 35 | 2 (4%) |
| HPV 39 | 2 (4%) | HPV 39 | 2 (4%) |
| HPV 73 | 2 (4%) | HPV 73 | 2 (4%) |
| HPV 31 | 2 (4%) | HPV 31 | 1 (2%) |
| HPV 33 | 1 (2%) | HPV 33 | 1 (2%) |
| **Among those with two HPV types (N=18)** | | | |
| Self-collection (N=14) | | Speculum collection (N=15) | |
| **HPV types** | **N (%)** | **HPV types** | **N (%)** |
| 16/59 | 3 (21%) | 16/59 | 3 (20%) |
| 16/39 | 1 (7%) | 16/39 | 1 (7%) |
| 16/52 | 1 (7%) | 16/18 | 2 (13%) |
|  |  | 16/56 | 1 (7%) |
| 39/68 | 2 (14%) | 39/68 | 1 (7%) |
| 39/52 | 1 (7%) | 39/52 | 1 (7%) |
|  |  | 39/56 | 1 (7%) |
| 33/56 | 1 (7%) | 56/68 | 1 (7%) |
| 31/56 | 2 (14%) | 31/56 | 1 (7%) |
| 59/68 | 1 (7%) | 59/68 | 1 (7%) |
| 45/68 | 1 (7%) | 45/68 | 1 (7%) |
| 18/45 | 1 (7%) | 18/45 | 1 (7%) |
| **Among those with three or more HPV types (N=6)** | | | |
| Self-collection (N=5) | | Speculum collection (N=4) | |
| **HPV types** |  | **HPV** **types** |  |
| 16/39/68 | 1 (20%) | 35/39/45 | 1 (25%) |
| 35/39/45 | 1 (20%) | 16/35/39/68 | 1 (25%) |
| 16/35/39/68 | 1 (20%) | 18/39/68/73 | 1 (25%) |
| 45/52/68/73 | 1 (20%) | 16/18/31/56/59/66/68/73 | 1 (25%) |
| 16/18/31/56/59/66/68/73 | 1 (20%) |  |  |

ƚ HPV means human papillomavirus
