# Supplementary material for: Cervical cancer screening: Impact of collection technique on human papillomavirus detection and genotyping
Source: Prev Med Rep. 2025 Jan 17;50:102971. doi: 10.1016/j.pmedr.2025.102971 (PMC11791345; doi:10.1016/j.pmedr.2025.102971)
Supplement: Supplementary file 3 — Supplementary material 3 [file mmc3.docx]

Supplementary Table 2 – Number of Human Papillomavirus types/US enrolled participants by high vs average risk cohort and collection technique, 2020-2022

|  | Number of HPVƚ types/person | | | |
| --- | --- | --- | --- | --- |
|  | **Zero** | **One** | **Two** | **Three or more** |
|  | **N (%)** | **N (%)** | **N (%)** | **N (%)** |
| **Total population**  **Self/Speculum (N=193)** | 116 (60%) | 53 (27%) | 18 (9%) | 6 (3%) |
| Colposcopy  Self/Speculum (N=97) | 33 (34%) | 43 (44%) | 16 (16%) | 5 (5%) |
| Primary Care Screening  Self/Speculum (N=96) | 83 (86%) | 10 (10%) | 2 (2%) | **1 (1%)** |
| **Total population Self-sample (N=193)** | **123 (64%)** | **51 (26%)** | **14 (7%)** | **5 (3%)** |
| Colposcopy  Self sample (N=97) | 39 (40%) | 42 (43%) | 12 (12%) | 4 (4%) |
| Primary Care Screening  Self-sample (N=96) | 84 (88%) | 9 (9%) | 2 (2%) | 1 (1%) |
| **Total population Speculum sample (N=193)** | **122 (63%)** | **50 (26%)** | **17 (9%)** | **4 (2%)** |
| Colposcopy  Speculum sample (N=97) | 36 (37%) | 42 (43%) | 16 (16%) | 3 (3%) |
| Primary Care Screening  Speculum sample (N=96) | 86 (90%) | 8 (8%) | 1 (1%) | 1 (1%) |

The total population means the colposcopy and primary care screening cohorts.

The collection technique means either a self-sampled vaginal specimen or a speculum-based cervical specimen.

Percentages may not add up to 100% due to rounding.

ƚHPV means human papillomavirus
