# Supplementary material for: Cervical cancer screening: Impact of collection technique on human papillomavirus detection and genotyping
Source: Prev Med Rep. 2025 Jan 17;50:102971. doi: 10.1016/j.pmedr.2025.102971 (PMC11791345; doi:10.1016/j.pmedr.2025.102971)
Supplement: Supplementary file 4 — Supplementary material 4 [file mmc4.docx]

Supplementary Table 3. Test Characteristics of the self and speculum collection techniques for the presence and absence of Cervical Intraepithelial Neoplasia Grade 2 or worse disease among a US high and average risk population, 2020-2022

| HPV ƚ Genotype | Positive Predictive Value(95% CI) | | Negative Predictive Value(95% CI) | | False Positive Rate  (95% CI) | | False Negative Rate  (95% CI) | | **False Positive Ratio**  **(95% CI)** | **False Negative Ratio(95% CI)** |
| --- | --- | --- | --- | --- | --- | --- | --- | --- | --- | --- |
|  | self collect | speculum collect | self collect | speculum collect | self collect | speculum collect | self collect | speculum collect | self/  speculum | self/  speculum |
| Overall | 0.16  (0.07, 0.25) | 0.16  (0.07, 0.25) | **1.00**  **(1.00, 1.00)** | **1.00**  **(1.00, 1.00)** | 0.58  (0.48, 0.69) | 0.61  (0.51, 0.71) | 0  (0, 0) | 0  (0, 0) | 0.96  (0.60, 1.56) | - |
| HPV 16 | 0.18  (0.02, 0.34) | 0.18  (0.02, 0.34) | 0.92  (0.86, 0.98) | 0.92  (0.86, 0.98) | 0.20  (0.12, 0.29) | 0.20  (0.12, 0.29) | 0.60  (0.30, 0.90) | 0.60  (0.30, 0.90) | 1.00  (0.49, 2.05) | 1.00  (0.24, 4.18) |
| HPV 18 | 0.20  (0, 0.55) | 0.11  (0, 0.32) | 0.90  (0.84, 0.96) | 0.90  (0.84, 0.96) | 0.04  (0.002, 0.09) | 0.09  (0.03, 0.15) | 0.90  (0.71, 1.00) | 0.90  (0.71, 1.00) | 0.50  (0.15, 1.72) | 1.00  (0.28, 3.57) |
| HPV 31 | 0  (0, 0) | 0  (0, 0) | 0.90  (0.83, 0.96) | 0.90  (0.83, 0.96) | 0.03  (0, 0.07) | 0.03  (0, 0.07) | 1.00  (1.00, 1.00) | 1.00  (1.00, 1.00) | 1.00  (0.20, 5.09) | 1.00  (0.29, 3.45) |
| HPV 33 | 0  (0, 0) | 0  (0, 0) | 0.90  (0.83, 0.96) | 0.90  (0.84, 0.96) | 0.02  (0, 0.05) | 0.01  (0, 0.03) | 1.00  (1.00, 1.00) | 1.00  (1.00, 1.00) | 2.00  (0.18, 22.46) | 1.00  (0.29, 3.45) |
| HPV 35 | 0.25  (0, 0.67) | 0.25  (0, 0.67) | 0.91  (0.85, 0.96) | 0.91  (0.85, 0.96) | 0.03  (0, 0.07) | 0.03  (0, 0.07) | 0.90  (0.71, 1.00) | 0.90  (0.71, 1.00) | 1.00  (0.20,5.09) | 1.00  (0.28, 3.57) |
| HPV 39 | 0.11  (0, 0.32) | 0.10  (0, 0.29) | 0.90  (0.84, 0.96) | 0.90  (0.84, 0.96) | 0.09  (0.03, 0.15) | 0.10  (0.04, 0.16) | 0.90  (0.71, 1.00) | 0.90  (0.71, 1.00) | 0.89  (0.33, 2.41) | 1.00  (0.28, 3.57) |
| HPV 45 | 0.29  (0, 0.62) | 0.38  (0.04, 0.71) | 0.91  (0.86, 0.97) | 0.92  (0.87, 0.98) | 0.06  (0.008, 0.10) | 0.06  (0.008, 0.10) | 0.80  (0.55, 1.00) | 0.70  (0.42, 0.98) | 1.00  (0.28, 3.57) | 1.14  (0.30, 4.37) |
| HPV 51 | 0.33  (0, 0.87) | 0  (0, 0) | 0.90  (0.84, 0.96) | 0.90  (0.84, 09.6) | 0.02  (0, 0.05) | 0.01  (0, 0.03) | 0.90  (0.71, 1.00) | 1.00  (1.00, 1.00) | 2.00  (0.18, 22.46) | 0.90  (0.26, 3.16) |
| HPV 52 | 0  (0, 0) | 0  (0, 0) | 0.89  (0.83, 0.96) | 0.89  (0.83, 0.96) | 0.07  (0.02, 0.12) | 0.07  (0.02, 0.12) | 1.00  (1.00, 1.00) | 1.00  (1.00, 1.00) | 1.00  (0.31, 3.22) | 1.00  (0.29, 3.45) |
| HPV 56 | 0  (0, 0) | 0  (0, 0) | 0.90  (0.83, 0.96) | 0.89  (0.83, 0.96) | 0.03  (0, 0.07) | 0.06  (0.008, 0.10) | 1.00  (1.00, 1.00) | 1.00  (1.00, 1.00) | 0.60  (0.14, 2.59) | 1.00  (0.29, 3.45) |
| HPV 58 | 0.25  (0, 0.67) | 0.33  (0, 0.87) | 0.91  (0.85, 0.96) | 0.91  (0.85 ,0.97) | 0.03  (0, 0.07) | 0.02  (0, 0.05) | 0.90  (0.71, 1.00) | 0.90  (0.71, 1.00) | 1.50  (0.24, 9.19) | 1.00  (0.28, 3.57) |
| HPV 59 | 0  (0, 0) | 0  (0, 0) | 0.89  (0.83, 0.96) | 0.89  (0.83, 0.96) | 0.06  (0.008, 0.10) | 0.06  (0.008, 0.10) | 1.00  (1.00, 1.00) | 1.00  (1.00, 1.00) | 1.00  (0.28, 3.57) | 1.00  (0.29, 3.45) |
| HPV 66 | 0(  0, 0) | 0  (0, 0) | 0.89  (0.83, 0.96) | 0.89  (0.83, 0.96) | 0.04  (0.002, 0.09) | 0.06  (0.008, 0.10) | 1.00  (1.00, 1.00) | 1.00  (1.00, 1.00) | 0.80  (0.21, 3.08) | 1.00  (0.29, 3.45) |
| HPV 68 | 0.11  (0, 0.32) | 0.11  (0, 0.31) | 0.90  (0.84, 0.96) | 0.90  (0.84, 0.96) | 0.09  (0.03, 0.15) | 0.09  (0.03, 0.15) | 0.90  (0.71, 1.00) | 0.90  (0.71, 1.00) | 1.00  (0.36, 2.78) | 1.00  (0.28, 3.57) |
| HPV 73 | 0  (0, 0) | 0  (0, 0) | 0.89  (0.83, 0.96) | 0.89  (0.83, 0.96) | 0.04  (0.002, 0.09) | 0.04  (0.002, 0.09) | 1.00  (1.00, 1.00) | 1.00  (1.00, 1.00) | 1.00  (0.24, 4.12) | 1.00  (0.29, 3.45) |

ƚ HPV means human papillomavirus
